# Imputing rare variants in families using a two-stage approach

**DOI:** 10.1186/s12919-016-0032-y

**Published:** 2016-10-18

**Authors:** Samantha Lent, Xuan Deng, L. Adrienne Cupples, Kathryn L. Lunetta, CT Liu, Yanhua Zhou

**Affiliations:** Department of Biostatistics, Boston University, Boston, MA USA

## Abstract

**Background:**

Recent focus on studying rare variants makes imputation accuracy of rare variants an important issue. Many approaches have been proposed to increase imputation accuracy among rare variants, from reference panel selection to combinations of existing methods to multistage analyses. We aimed to bring the strengths of these new approaches together with our proposed two-stage imputation for family data.

**Methods:**

Our imputation methods were tested on the region from 46.75Mb to 49.25Mb on chromosome 3. We did quality control based on the proportion of missing genotypes per variant and individual, leaving 495 individuals with 761 genome-wide association studies (GWAS) variants only, 45 with 14,077 sequence variants only, and 419 with both GWAS and sequencing data. All data were prephased using SHAPEIT2 with a duo hidden Markov model algorithm prior to performing imputation. Imputations were performed 100 times, each time masking the sequence data for 1 individual and imputing it from the GWAS data. We used well-imputed genotypes, defined as a probability of greater than 0.9, above 2 different minor allele frequency cutoffs—0.01 and 0.05—from Impute2 as input for Merlin, and compared these results to Impute2 and Merlin separately. The imputed results were evaluated using correlation measurement and the imputation quality score.

**Results:**

Our method improved imputation accuracy, measured by imputation quality score, for variants with minor allele frequency between 0.01 and 0.40, but failed to improve accuracy for variants with minor allele frequency less than 0.01 when we used a minor allele frequency cutoff of 0.01 for the Impute2 results. In contrast, our 2-stage approach with a minor allele frequency cutoff of 0.05 performed the worst of all methods for variants with minor allele frequency between 0.01 and 0.40.

**Conclusions:**

This method gave promising results, but may be further improved by changing the inclusion criteria of Impute2 variants. More analyses are needed on a larger region with different inclusion thresholds to assess the accuracy of this approach.

## Background

Although existing population-based genotype imputation methods are very accurate for common variants, with overall best-guess error rates of 5 % to 7 % for the most common methods [1], they do not perform nearly as well with rare variants. Only 78 % of variants with a minor allele frequency (MAF) between 0.01 and 0.05 in the Illumina 550K panel and 57 % in the Affymetrix 500K panel can be well imputed (r^2^ > 0.7) using BEAGLE [2].

Most efforts to improve rare variant imputation have focused on how the choice of reference panel affects imputation quality. However, recently Saad et al [3] and Kreiner-Møller et al [4] have proposed methods to improve imputation using multistep procedures. Saad et al proposed using 2 imputation methods independently, 1 population based (BEAGLE) and 1 family based (Genotype Imputation Given Inheritance [GIGI]), and choosing the imputed data from the method with the highest variance in genotype probabilities for each single nucleotide polymorphism (SNP). For instance, if the probabilities for genotypes AA, AB, and BB in an individual are 0, 0, and 1.0, respectively, for BEAGLE and 0, 0.5, and 0.5 for GIGI, Saad et al’s method would choose BEAGLE for that variant, because the larger variance indicates more certainty in the call. Saad et al found that the combined method led to more accurate imputed genotypes than either method separately. Kreiner-Møller et al suggested a 2-step imputation using a local reference panel and the 1000 Genomes reference panel, implemented in MACH/Minimac [4, 5]. In the first step, they imputed the study sample to a densely genotyped local reference panel enriched for rare variants. Next, they used the best-guess genotypes from this imputation as well as the original genotypes to impute the study sample to the 1000 Genomes panel.

Our approach combined the strengths of Saad et al and Kreiner-Møller et al. We performed a 2-stage imputation, implementing Impute2 and Merlin sequentially, to test the hypothesis that increasing the density of genotypes in a sequenced reference panel using a population-based imputation before performing a family-based imputation would lead to higher imputation accuracy in a related genome-wide association studies (GWAS) study panel.

## Methods

### Quality control

Our sample consisted of 959 Mexican Americans from 20 families. All 959 subjects were genotyped on the Illumina platform, and 464 of these individuals were also sequenced. We removed all SNPs with more than 5 % missing data and all individuals with more than 5 % missing data (*N =* 45) from the GWAS samples, and limited our analysis to the 46.75 Mb to 49.25 Mb region on chromosome 3. This yielded 914 people with GWAS data and 761 Illumina variants. For the sequenced data, we removed any variant with more than 10 % missing data, leaving 14,077 sequenced variants. All sequenced individuals had less than 5 % missing data. Thus, all 959 individuals were included in the analyses: 495 with GWAS only, 45 with sequencing only, and 419 with both GWAS and sequencing.

### Phasing

All data were prephased using SHAPEIT2 prior to performing imputation [6]. We used the duo hidden Markov model (duoHMM) algorithm in SHAPEIT, which uses pedigree information from trios to improve phasing and eliminate Mendelian errors. GWAS and sequence data were phased in separate runs.

### Imputation

We performed 100 imputations each with 3 different methods: population-based imputation with Impute2 2.3.1, family-based imputation with Merlin 1.1.2, and a combination of the two [7, 8]. For each of these 100 imputations, we masked the sequence data of 1 individual, using the individual’s GWAS data instead, and imputed the sequenced variants not in the GWAS data. After the imputation, we compared this individual’s imputed genotypes to his or her true sequenced genotypes. We chose which sequenced subjects to leave out by randomly ordering all 419 subject IDs—excluding the 45 participants with sequence data but no GWAS data—and choosing the first 100.

For the population-based imputation benchmark, we used Impute2 with the default settings. The reference panel included both a local reference panel of the sequenced study individuals and a cosmopolitan reference panel of all populations from the 1000 Genomes Project (1KGP) [5]. For the family-based imputation benchmark, we used Merlin, which combines sparse marker data and high-density genotype data on several individuals to infer unobserved high-density genotypes for related individuals [9]. In the Merlin-only imputation, only our population samples were used as the imputation backbone. Each Merlin imputation included the masked individual and their nuclear family, grandchildren, and grandparents. Table 1 shows the distribution of family size for 100 individuals. The maximum proportion of parents and spouses of the masked individuals with genotype data for sequence variants is 0.667 and the minimum proportion is 0. The mean proportion is 0.3796 with a standard deviation of 0.35.

**Table 1:** Distribution of family size

Family size	3	4	5	6	7	8	9	10	11	12	13	14	15
No. of families	6	8	24	17	15	5	8	1	3	7	2	2	2

Because the algorithm used in Merlin depends on markers being in linkage equilibrium (LE), we also compared the family-based imputation qualities by using sparse markers, dense markers, or the haplotype-block approach [10] (with –cluster option in Merlin). To get sparse markers, we pruned the GWAS variants in the region (46.75Mb to 49.25Mb) on chromosome 3 by only keeping variants with pairwise *r* ^2^ less than 0.2 implemented in PLINK 1.9, which yielded 91 variants in approximate LE. The mean pairwise *r* ^2^ for the 91 variants was 0.0252 and the median was 0.0014. To get the clustered markers and haplotype frequencies, we searched for GWAS markers for which *r* ^2^ is larger than 0.2 and defined the clusters, including each identified pair and intervening markers, which were implemented in Merlin with the–rsq and –cfreq options. The imputations were conducted with all GWAS variants (dense markers), pruned GWAS variants in LE and dense markers with predefined haplotypes, separately. Table 2 presents the imputation quality measurements (correlation and imputation quality score [IQS]). Because of the slight differences between these 3 strategies as seen in Table 2 and the fraction of parents and spouses of the masked individuals having genotype data for sequence variants, we conclude that the linkage disequilibrium present in the data is not affecting the Merlin imputation adversely in this study.

**Table 2:** Summary statistics of correlation and IQScomparing the imputation with dense markers and sparse markers

Quality measurements		Minimum	Median	Mean	Maximum	SD
Correlation	Masked individuals with GWA	0.00049	0.6983	0.5766	1	0.3714
	Masked individuals with GWA in LE	0.00087	0.6798	0.5708	1	0.3729
	Impute with –cluster option	0.0000	0.6879	0.5748	1	0.3727
IQS	Masked individuals with GWA	−0.04793	0.4046	0.3758	0.9715	0.3007
	Masked individuals with GWA in LE	−0.04758	0.3883	0.3659	0.9682	0.2980
	Impute with –cluster option	−0.04887	0.3989	0.3712	0.9682	0.2995

*GWA* genome-wide association

Finally, for the combined imputation method, we selected the best-guess genotypes for all SNPs with MAF greater than 2 different cutoffs—0.01 and 0.05—and posterior probability of the best-guess genotype greater than 0.9, and used these genotypes as well as the GWASSNPs as input for Merlin. Merlin automatically excluded from imputation any variant with Mendelian-inconsistent genotyping errors, but it is possible that Impute2 introduced Mendelian-consistent genotyping errors. However, the 2-stage and Merlin-only results were almost identical for variants with MAFs below the cutoff, which leads us to believe that these potential errors introduced by Impute2 did not negatively affect imputation quality in our sample.

### Accuracy assessment

We used 2 different measures of accuracy: correlation between imputed dosage and true dosage and IQS, a measure developed by Lin et al in 2010 [11], inspired by Cohen’s Kappa statistic [12]. Cohen’s Kappa measures the agreement between 2methods of classification, adjusting for chance agreement. To apply this to imputation results, we first tabulate the imputed best-guess genotypes and true genotypes, as shown in Table 3, where n_ij_ is the number of individuals with true genotype i and imputed genotype j. Cohen’s Kappa statistic is given by:

$$ \kappa =\frac{\frac{{\displaystyle {\sum}_i}{n}_{ii}}{n_{..}}-\frac{{\displaystyle {\sum}_i}{n}_{i.}{n}_{.i}}{n_{..}^2}}{1-\frac{{\displaystyle {\sum}_i}{n}_{i.}{n}_{.i}}{n_{..}^2}} $$

**Table 3:** Tabulation of genotypes used for IQS calculation

	True Genotypes			
Imputed Genotypes	AA	AB	BB	Total
AA	n_11_	n_12_	n_13_	n_1._
AB	n_21_	n_22_	n_23_	n_2._
BB	n_31_	n_32_	n_33_	n_3._
Total	n_.1_	n_.2_	n_.3_	n..

This statistic adjusts for agreement by chance by subtracting the expected cell counts along the diagonal (which indicates agreement) from the observed proportion of agreement. In cases where the expected agreement is high, such as with variants with low MAFs, the second term in the numerator is higher, thus lowering the Kappa statistic. Lin et al extended this idea to incorporate the uncertainty of imputation by using the posterior probabilities of all 3 genotypes instead of the best-guess genotype, thus allowing the cells in Table 3 to have noninteger values. Cohen’s Kappa and the IQS are equivalent when all cells in Table 3 are integers (ie, when all posterior probabilities are 0 or 1), but differ when there is uncertainty in the imputation. Consequently, IQS is useful for rare variants because, unlike concordance, it accounts for allele frequency and adjusts for chance agreement. Furthermore, IQS can be computed using dosages, which gives more information about imputation quality than best-guess genotypes. Lin et al have compared the performance of IQS and concordance for population-based imputations implemented in Impute2. The authors show that concordance increases with decreased MAF, whereas IQS drops as MAF decreases. The decreasing imputation quality with decreasing MAF is expected, as rare variants do not impute well [13], making IQS a better measure of imputation quality.

## Results

Among 100 individuals that we selected, the number of imputed polymorphic sequence variants is 6726. The accuracy assessments with IQS and correlation were conducted within the 100 individuals and polymorphic variants. However, different imputation strategies yield different numbers of polymorphic variants with meaningful IQS or correlation (Table 4). This is because both imputed and true genotypes must be polymorphic to obtain a meaningful IQS or correlation, and the number of polymorphic imputed genotypes varied by method.

**Table 4:** Summary of Imputation Quality by MAF

Imputation Approach	(0,0.01) 4028 SNPs			(0.01,0.05) 1416 SNPs			(0.05,0.4) 1142 SNPs		
	#SNPp*	Mean	Var	#SNPp*	Mean	Var	#SNPp*	Mean	Var
IQS									
Impute2	3023	0.840	0.097	1164	0.772	0.126	761	0.899	0.048
Merlin	4028	0.348	0.132	1416	0.437	0.041	1142	0.404	0.006
Combined (0.01)^a^	4028	0.350	0.133	1416	0.965	0.017	1142	0.992	0.004
Combined (0.05) ^a^	4028	0.349	0.133	1416	0.443	0.041	1142	0.992	0.004
Correlation									
Impute2	3023	0.918	0.048	1164	0.981	0.006	761	0.999	0.00004
Merlin	4028	0.512	0.195	1416	0.663	0.064	1142	0.687	0.007
Combined (0.01) ^a^	4028	0.514	0.196	1416	0.975	0.010	1142	0.994	0.003
Combined (0.05) ^a^	4028	0.513	0.196	1416	0.669	0.063	1142	0.993	0.003

*#SNP_p_ is the number of SNPs with a MAF greater than 0 for both real and imputed genotypes (varies by method)

^a^Combined (m) indicates the 2-stage imputation approach with MAF cutoff m

**Fig. 1:**
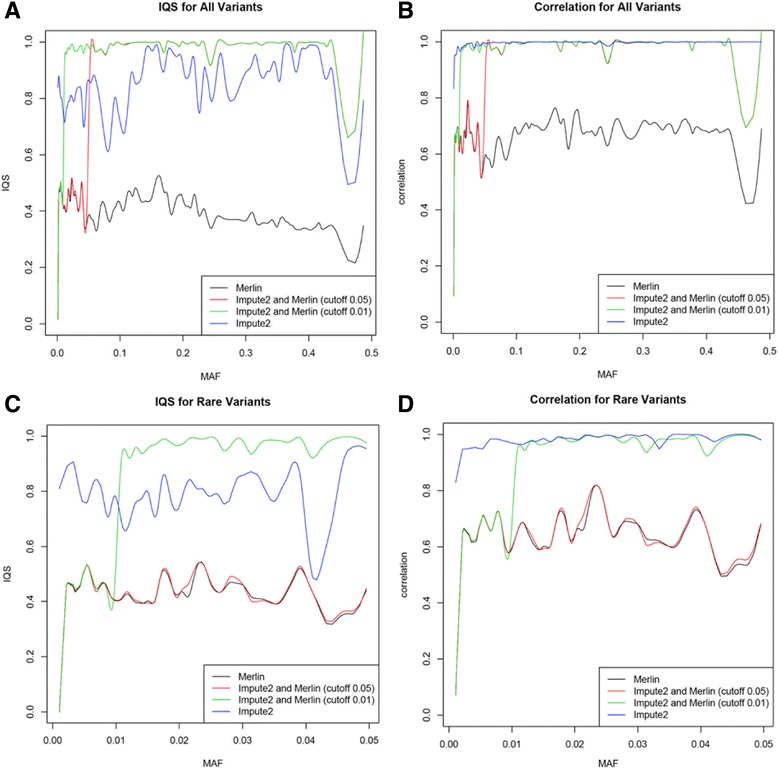
Imputation quality vs. MAF. **a** IQS for all polymorphic sequence variants. **b** Correlation between true and imputed dosages for all polymorphic sequence variants. **c** IQS for rare (MAF < 0.05) polymorphic sequence variants. **d** Correlation between true and imputed dosages for rare (MAF < 0.05) polymorphic sequence variants

Generally, our proposed 2-step imputation method performed better than only using population-based imputation with Impute2 or only using family-based imputation with Merlin for the variants with a MAF larger than 0.1 and less than 0.4 (Figs. 1a and b). With decreasing the cutoff of MAF for selected imputed variants from population-based imputation using Impute2, the imputation of our method outperformed for most of rare variants with minor MAF between 0.01 and 0.05 (Figs. 1c and d). For common variants, the different cutoffs of the MAFs give similar imputations.

## Discussion

Our combined method with a MAF cutoff of 0.01 performed better than either Merlin or Impute2 alone for variants with MAFs between 0.01 and 0.4, and our combined method with a MAF cutoff of 0.05 performed better than either Merlin or Impute2 alone for variants with MAFs >0.05. Because the performance suffers below our MAF cutoffs, this suggests that we should not filter Impute2 results by MAF at all, but filter only by best-guess genotype probability.

One potential limitation of this study is that families with more sequence data were more likely to be selected in our set of 100 individuals. We would expect higher imputation accuracy in these families, as there were more individuals included in the reference panels for imputation. More work needs to be done to determine exactly how much the number and relationships of sequenced family members available affect imputation quality. This was beyond the scope of our project, but may be useful in helping investigators choose which family members to sequence.

It is unclear from these results whether the sequential nature of the imputation increases accuracy. In the future, we should compare our method to a method combining independent results from Merlin and Impute2, both based on best-guess genotype probability and Saad et al’s proposed vote strategy [3]. Furthermore, future studies should be done on a larger region and larger sample size, and potentially include different probability thresholds for the Impute2 results.

## Conclusions

Our 2-stage method with a MAF inclusion cutoff of 0.01 for Impute2 results achieved better IQSs than either Impute2 or Merlin alone, and similar correlation values, for variants with MAFs between 0.01 and 0.4. This method could be further improved by including all Impute2 imputed genotypes above a certain quality threshold regardless of MAF. Other probability thresholds should be tested, and this 2-stage method should be compared to results using Merlin and Impute2 independently to examine whether the sequential nature of the procedure increases accuracy above and beyond the increase obtained by combining population- and family-based methods.
